# The complete mitochondrial genome and phylogenetic analysis of *Anabarilius duoyiheensis* Li, Mao & Lu, 2002 (Cypriniformes: Xenocyprididae)

**DOI:** 10.1080/23802359.2023.2254459

**Published:** 2023-09-20

**Authors:** Junjie Wu, Fangpeng Jin, Jingxia Zhao, Hongman Yu, Zhifei Wang, Xing Liu, Pengxiang Zuo, Jianyu Song, Xiangxing Lu, Yun Leng

**Affiliations:** a Yunnan Institute of Fishery Sciences Research, Kunming, China; b Key Laboratory of Yunnan Characteristic Fish Protection and Germplasm Innovation, Kunming, China; c College of Agronomy and Biotechnology, Yunnan Agricultural University, Kunming, China; d Lubuge Township Fisheries Station, Qujing, China

**Keywords:** *Anabarilius duoyiheensis*, mitochondrial genome, phylogenetic analysis

## Abstract

*Anabarilius duoyiheensis* is a native and rare fish in Yunnan. In this study, the complete mitochondrial genome of *A. duoyiheensis* was sequenced and published for a total of 16,614 bp, including 13 protein-coding genes, 22 transfer RNAs, two ribosomal RNAs, and one control region. The phylogenetic analysis based on the complete mitochondrial genome showed that *A. duoyiheensis* belongs to the clade of the genus *Anabarilius* and was sister to the clade of *Hemiculter*. This study also contributes to the genus phylogeny of *Anabarilius* and other members of the family Xenocyprididae.

## 1. Introduction

*Anabarilius duoyiheensis* Li, Mao & Lu, 2002 belongs to Cypriniformes, Xenocyprididae, *Anabarilius* and distributed in Duoyi river in Qujing, Yunnan, China (Li et al. 2002; Chen X 2013). The morphological features of *A. duoyiheensis* were: mouth terminal; head length is significantly greater than body height; arcuate abdomen; incomplete ventral edge; long pectoral fins with terminal tip; short branchial rake (Li et al. 2002). Some of the main features were similar to those of *A. macrolepis* with distinguishable features: fewer branchial rake (7–8); the end of unbranched dorsal fin is hard; longer pectoral fin (Li et al. 2002). The *Anabarilius* species, distributed mainly in central and eastern Yunnan and south of Sichuan Province, were peculiar to China (Yang J and Chu 1987). However, there are no data regarding *A. duoyiheensis* and particularly its mitochondrial genome. Here, we presented the mitochondrial genome of *A. duoyiheensis* by using next-generation sequencing, and these results will be useful in further phylogenetic analysis of genus of *Anabarilius.*

## 2. Materials and methods

### 2.1. Specimens collection and preservation

**Figure 1.**
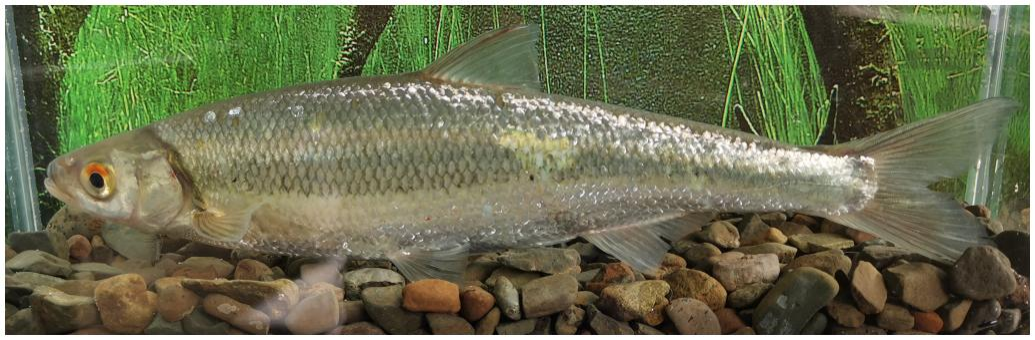
Specimen of *Anabarilius duoyiheensis* was collected in the Duoyi river, Yunnan Province, China. Photographed by Yun Leng on 16 November 2021.

The biological specimens of *A. duoyiheensis* were sampled from the Lu Buge reach of the Duoyi river (N: 24°45.669′, E: 104°29.758′) in Qujing, Yunnan, and the live fish were kept in Lubuge Township Fisheries Station. A specimen was deposited at Yunnan Institute of Fishery Sciences Research with the specimen voucher number: DYBY-01 (contact person: Junjie Wu, wujunjie2007@yeah.net). The specimen of *A. duoyiheensis* was identified by morphological feature according to Li’s description (Li et al. 2002), and descriptive image was taken by Leng (corresponding author) (Figure 1).

### 2.2. Illumina sequencing, assembly, and annotation

The tissue samples were homogenized and DNA was extracted by following ammonium acetate precipitation method (Rivero et al. 2006). The Library was prepared according to Illumina protocol, and sequenced in Illumina NovaSeq platform (Modi et al. 2021). The contigs were assembled using SPAdes V3.13 with -k 127 (Bankevich et al. 2012). The functional annotation of mitochondrial genome was performed using MITOS2 (http://mitos2.bioinf.uni-leipzig.de/index.py) (Donath et al. 2019).

### 2.3. Phylogenetic analysis

To analyze the phylogenetic relationship of *A. duoyiheensis*, published mitochondrial sequences of other 18 species in the family of Xenocyprididae were chosen; the species information and references are listed in Table 1. The alignment sequences were obtained in MEGA X using MUSCL method (Kumar et al. 2008), and substitution model was evaluated in jModeltest 2.1.3 (Posada 2008). The AICc results were carried in phyML 3.0 for rebuilding the maximum-likelihood (ML) tree (Guindon et al. 2009). *S. curriculus* (Zeng S et al. 2016) was chosen and regarded as outgroup.

**Table 1.** Whole mitochondrial genomes used to infer the phylogenetic analysis.

Genus	Species name	Size (bp)	GenBank accession number	Reference
*Anabarilius*	*Anabarilius brevianalis* (Zhou & Cui, 1992)	16,610	MK757491.1	He Y et al. (2019)
	*Anabarilius duoyiheensis* (Li, Mao & Lu, 2002)	16,614	OM731672.1	This study
	*Anabarilius liui yalongensis* (Chang, 1944)	16,608	MG702493.1	Zeng L et al. (2019)
*Hemiculterella*	*Hemiculterella sauvagei* (Warpachowski, 1888)	16,618	KP316066.1	He B et al. (2016)
*Hemiculter*	*Hemiculter leucisculus* (Basilewsky, 1855)	16,621	AP012110.1	Luo et al. (2022)
	*Hemiculter tchangi* (Fang, 1942)	16,621	MG554419.1	Zou et al. (2019)
	*Hemiculter eigenmanni* (Basilewsky, 1855)	16,626	KT952322.1	–
	*Hemiculter bleekeri bleekeri* (Warpachowski, 1888)	16,617	KT361083.1	Dai et al. (2016)
*Megalobrama*	*Megalobrama skolkovii* (Basilewsky, 1855)	16,620	KJ630486.1	Yang H, Li, et al. 2016
	*Megalobrama pellegrini* (Tchang, 1930)	16,620	KP262030.1	Liu X et al. (2016)
	*Megalobrama amblycephala* (Yih, 1955)	16,623	AP011219.1	Guo et al. (2017)
	*Megalobrama terminalis* (Richardson, 1846)	16,623	AB626850.1	Imoto et al. (2013)
*Culter*	*Culter oxycephaloides* (Kreyenberg & Pappenheim, 1908)	16,619	KY404014.1	Chen H et al. (2019)
	*Culter mongolicus* (Basilewsky, 1855)	16,622	AP009060.1	Saitoh et al. (2006)
	*Culter recurviceps* (Richardson, 1846)	16,622	KJ609181.1	Yang H, Zhao, et al. (2016)
	*Culter alburnus* (Basilewsky, 1855)	16,622	AP012109.1	Liu K et al. (2020)
*Chanodichthys*	*Chanodichthys ilishaeformis* (Bleeker, 1871)	16,624	KU200257.1	Li S et al. (2016)
	*Chanodichthys erythropterus* (Basilewsky, 1855)	16,623	MN105126.1	Chen L et al. (2016)
*Squaliobarbus*	*Squaliobarbus curriculus* (Richardson, 1846)	16,622	KP731975.1	Zeng S et al. (2016)

## 3. Results

### 3.1. Mitochondrial characterization

**Figure 2.**
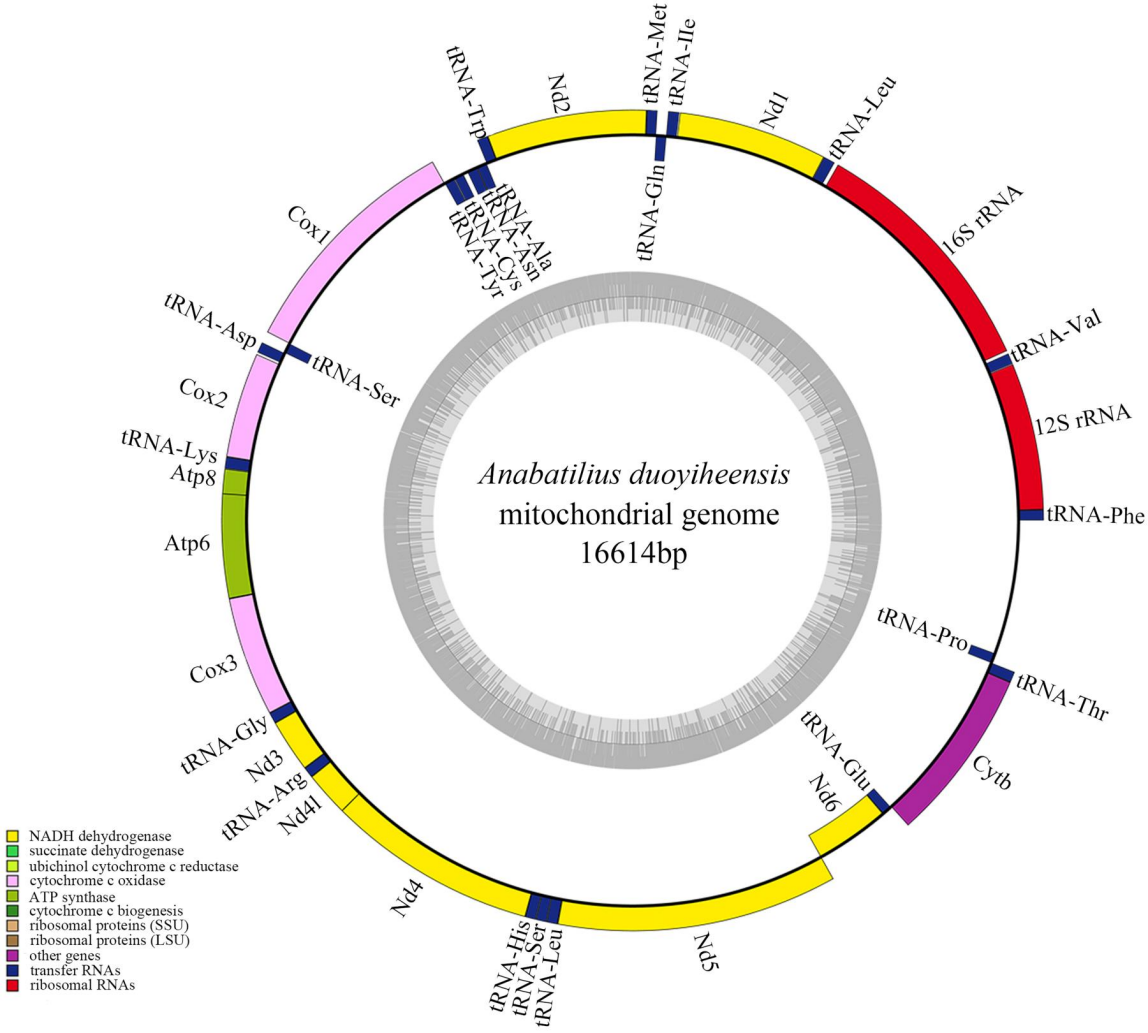
Complete mitochondrial genome map of *A. duoyiheensis* (GenBank: OM731672.1), with 13 genes, 22 tRNAs, and two rRNAs. Encoded genes and RNAs were in different colors; light and heavy strands were shown inside and outside of the circle, respectively.

The complete mitochondrial DNA sequence of *A. duoyiheensis* was 16,614 bp in length with a depth of 582.42x (Figure S1, Table S1), including 13 protein-coding genes, 22 transfer RNAs (tRNAs), two ribosomal RNAs (rRNAs), and a D-loop control region (Figure 2). The initiation codon of 13 protein coding genes began with ATG except COX1, which began with GTG. Incomplete stop codons were identified in Nd2, Cox2, Cox3, Nd3, Nd6, and Cytb; TAA or TAG stop codon was identified in Nd1, Cox1, Atp8, Atp6, Nd4l, and Nd5. The base composition was 28.87%A, 24.89%T, 27.86%C, and 18.37%G, the A + T content was 53.76% and G + C content was 46.24%. Most of the genes were in the heavy strand (H-strand), except for ND6 and eight tRNAs. The blast search shows that the identity of *A. duoyiheensis* mitochondrial sequences was 94% similar to *A. liui yalongensis*, 93% to *A. brevianalis*, and 91% to *H. leucisculus* and *H. tchangi*.

### 3.2. Phylogenetic analysis

**Figure 3.**
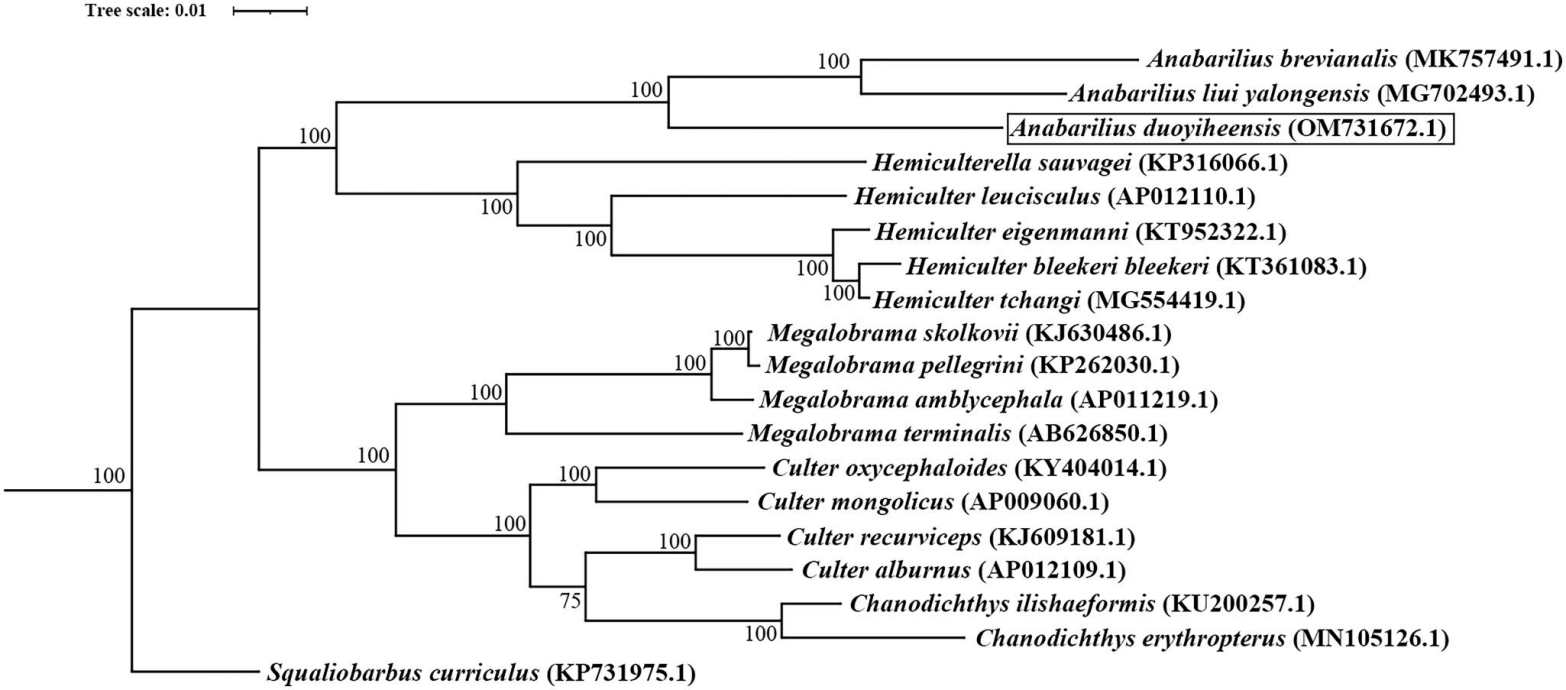
Phylogenetic tree of *A. duoyiheensis* and other 18 species based on the complete mitochondrial sequences. *Squaliobarbus curriculus* was set to be outgroup. The bootstrap values were marker near the nodes. Accession numbers for each species were shown after the name of the species. The *A. duoyiheensis* generated in this study (OM731672.1) was labeled in black outline.

The model of GTR + I + G was the perfect substitution model for phylogenetic construction. The ML tree showed that *A. duoyiheensis* is phylogenetically closest to the clade of *A. liui yalongensis* and *A. brevianalis.* The genus of *Anabarilius* was monophyletic, and a sister group to clade of genus *Hemiculter* (Figure 3).

## 4. Discussion and conclusions

In this study, we first presented the mitochondrial genome of *A. duoyiheensis*, which is also the first molecular biological data for this species. The availability of this mitochondrial genome will be essential for future DNA barcoding, taxonomic, and phylogenetic studies of the family Xenocyprididae, and also valuable for resource conservation.

## Supplementary Material

Supplemental Material

Supplemental Material

## Data Availability

The complete mitochondrial genome assembly data were available in GenBank database under the accession number OM731672.1 (https://www.ncbi.nlm.nih.gov/nuccore/OM731672.1/). The associated BioProject, BioSample, and SRA numbers are PRJNA909186, SAMN32069822, and SRR22548207, respectively.

## References

[CIT0001] Bankevich A, Nurk S, Antipov D, Gurevich AA, Dvorkin M, Kulikov AS, Lesin VM, Nikolenko SI, Pham S, Prjibelski AD, et al. 2012. SPAdes: a new genome assembly algorithm and its applications to single-cell sequencing. J Comput Biol. 19(5):455–477. doi: 10.1089/cmb.2012.0021. 22506599 PMC3342519

[CIT0002] Chen H, Wang D, Duan X, Liu S, Chen D. 2019. The mitochondrial genome of *Culter oxycephaloides* (Cypriniformes, Cyprinidae). Mitochondrial DNA B Resour. 4(2):2392–2393. doi: 10.1080/23802359.2017.1383196. 33365558 PMC7687434

[CIT0003] Chen L, Li B, Zhou L, Zhao G. 2016. The complete mitochondrial genome sequence of Predatory carp *Chanodichthys erythropterus* (Cypriniformes: Cyprinidae). Mitochondrial DNA A DNA Mapp Seq Anal. 27(2):1119–1120. doi: 10.3109/19401736.2014.933328. 25010069

[CIT0004] Chen X. 2013. Checklist of fishes of Yunnan. Dongwuxue Yanjiu. 34(4):281–343. doi: 10.11813/j.issn.0254-5853.2013.4.0281. 23913883

[CIT0005] Dai X, Li W, Tian S. 2016. Complete mitochondrial genome of *Hemiculter bleekeri bleekeri*. Mitochondrial DNA A DNA Mapp Seq Anal. 27(6):4338–4339. doi: 10.3109/19401736.2015.1089490. 26465980

[CIT0006] Donath A, Jühling F, Al-Arab M, Bernhart SH, Reinhardt F, Stadler PF, Middendorf M, Bernt M. 2019. Improved annotation of protein-coding genes boundaries in metazoan mitochondrial genomes. Nucleic Acids Res. 47(20):10543–10552. doi: 10.1093/nar/gkz833. 31584075 PMC6847864

[CIT0007] Guindon S, Delsuc F, Dufayard JF, Gascuel O. 2009. Estimating maximum likelihood phylogenies with PhyML. Methods Mol Biol. 537:113–137. doi: 10.1007/978-1-59745-251-9_6. 19378142

[CIT0008] Guo G, Yi S, Wang W. 2017. The complete mitochondrial genome of Yihe bream, *Megalobrama amblycephala* Yih. Mitochondrial DNA B Resour. 2(2):566–567. doi: 10.1080/23802359.2017.1365652. 33473902 PMC7800805

[CIT0009] He B, Chen Y, Liu Y, Du J, Deng X. 2016. The complete mitochondrial genome of *Hemiculterella sauvagei* (Teleostei, Cyprinidae, Hemiculterella). Mitochondrial DNA A DNA Mapp Seq Anal. 27(5):3322–3324. doi: 10.3109/19401736.2015.1018204. 25693700

[CIT0010] He Y, Gong J, Zhu T, Wu X, Zhu Y, Yang D. 2019. Complete mitochondrial genome and phylogenetic analysis of *Anabarilius brevianalis* (Teleostei, Cyprinidae, Cultrinae). Mitochondrial DNA Part B. 4(1):1834–1836. doi: 10.1080/23802359.2019.1612717.

[CIT0011] Imoto JM, Saitoh K, Sasaki T, Yonezawa T, Adachi J, Kartavtsev YP, Miya M, Nishida M, Hanzawa N. 2013. Phylogeny and biogeography of highly diverged freshwater fish species (Leuciscinae, Cyprinidae, Teleostei) inferred from mitochondrial genome analysis. Gene. 514(2):112–124. doi: 10.1016/j.gene.2012.10.019. 23174367

[CIT0012] Kumar S, Nei M, Dudley J, Tamura K. 2008. MEGA: a biologist-centric software for evolutionary analysis of DNA and protein sequences. Brief Bioinform. 9(4):299–306. doi: 10.1093/bib/bbn017. 18417537 PMC2562624

[CIT0013] Li S, Liu X, Bai Y, Huang T, Wang J, Qu H, Chen L, Jiang W. 2016. The complete mtDNA genome of *Erythroculter ilishaeformis*: genome characterization and phylogenetic analysis. Mitochondrial DNA B Resour. 1(1):39–40. doi: 10.1080/23802359.2015.1137812. 33473399 PMC7800875

[CIT0014] Li W, Mao W, Lu Z. 2002. A new species of Cyprinidae from Yunnan. J Zhanjiang Ocean Univ. 22(1):1–2.

[CIT0015] Liu K, Ma HJ, Feng XY, Xie N. 2020. Complete mitochondrial genome of the hybrid of *Culter alburnus* (♀) × *Megalobrama terminalis* (♂). Mitochondrial DNA B Resour. 5(3):2316–2317. doi: 10.1080/23802359.2020.1772690. 33457773 PMC7782101

[CIT0016] Liu X, Xiao K, Zhao X, Liu J, Zhang X, Guo W, Chen L, Du H. 2016. The complete mitochondrial genome of the *Megalobrama pellegrini* (Teleostei: Cyprinidae). Mitochondrial DNA A DNA Mapp Seq Anal. 27(5):3069–3070. doi: 10.3109/19401736.2014.1003913. 25600731

[CIT0017] Luo C, Chen P, Zhu J, Ma Z. 2022. The complete mitogenome of *Hemiculter leucisculus* (Basilewsky, 1855) in Hainan Island and its phylogenetic status. Mitochondrial DNA B Resour. 7(2):396–398. doi: 10.1080/23802359.2022.2040390. 35224195 PMC8865130

[CIT0018] Modi A, Vai S, Caramelli D, Lari M. 2021. The Illumina sequencing protocol and the NovaSeq 6000 system. Methods Mol Biol. 2242:15–42. doi: 10.1007/978-1-0716-1099-2_2. 33961215

[CIT0019] Posada D. 2008. jModelTest: phylogenetic model averaging. Mol Biol Evol. 25(7):1253–1256. doi: 10.1093/molbev/msn083. 18397919

[CIT0020] Rivero ER, Neves AC, Silva-Valenzuela MG, Sousa SO, Nunes FD. 2006. Simple salting-out method for DNA extraction from formalin-fixed, paraffin-embedded tissues. Pathol Res Pract. 202(7):523–529. doi: 10.1016/j.prp.2006.02.007. 16723190

[CIT0021] Saitoh K, Sado T, Mayden RL, Hanzawa N, Nakamura K, Nishida M, Miya M. 2006. Mitogenomic evolution and interrelationships of the Cypriniformes (Actinopterygii: Ostariophysi): the first evidence toward resolution of higher-level relationships of the world’s largest freshwater fish clade based on 59 whole mitogenome sequences. J Mol Evol. 63(6):826–841. doi: 10.1007/s00239-005-0293-y. 17086453

[CIT0022] Yang H, Li Q, Shu H, Yang L, Wu X, Yue L, Hou L. 2016. The complete mitochondrial genome of the *Megalobrama skolkovii* (Cyprinidae: Cultrinae). Mitochondrial DNA A DNA Mapp Seq Anal. 27(1):736–737. doi: 10.3109/19401736.2014.913165. 24810068

[CIT0023] Yang H, Zhao H, Sun J, Xie Z, Yang Z, Liu L. 2016. The complete mitochondrial genome of the *Culter recurviceps* (Teleostei, Cyprinidae). Mitochondrial DNA A DNA Mapp Seq Anal. 27(1):762–763. doi: 10.3109/19401736.2014.915529. 24810061

[CIT0024] Yang J, Chu X. 1987. Phylogeny of the Cyprinid genus Anabarilius (Pisces: Cyprinidae). Zool Res. 8(3):261–276.

[CIT0025] Zeng L, Yang K, Wen A, Xie M, Yao Y, Xu H, Zhu G, Wang Q, Jiang Y, He T, et al. 2019. Complete mitochondrial genome of the endangered fish *Anabarilius liui yalongensis* (Teleostei, Cyprinidae, Cultrinae). Mitochondrial DNA B Resour. 4(1):21–22. doi: 10.1080/23802359.2018.1535850. 33365403 PMC7510629

[CIT0026] Zeng S, Zhou L, Wang P, Tang Q, Zeng L, Xu P, Li G. 2016. The mitochondrial genome of *Squaliobarbus curriculus* of Pearl River. Mitochondrial DNA A DNA Mapp Seq Anal. 27(6):4217–4218. doi: 10.3109/19401736.2015.1022747. 25865851

[CIT0027] Zou Y, Liu T, Li Q, Wen Z, Qin C, Li R, Wang D. 2019. Complete mitochondrial genome of *Hemiculter tchangi* (Cypriniformes, Cyprinidae). Conserv Genet Resour. 11(1):1–4. doi: 10.1007/s12686-017-0949-0.

