# Supplementary figures and images for: The complete mitochondrial genome and phylogenetic analysis of Anabarilius duoyiheensis Li, Mao & Lu, 2002 (Cypriniformes: Xenocyprididae)
Source: Mitochondrial DNA B Resour. 2023 Sep 20;8(9):989–92. doi: 10.1080/23802359.2023.2254459 (PMC10512799; doi:10.1080/23802359.2023.2254459)

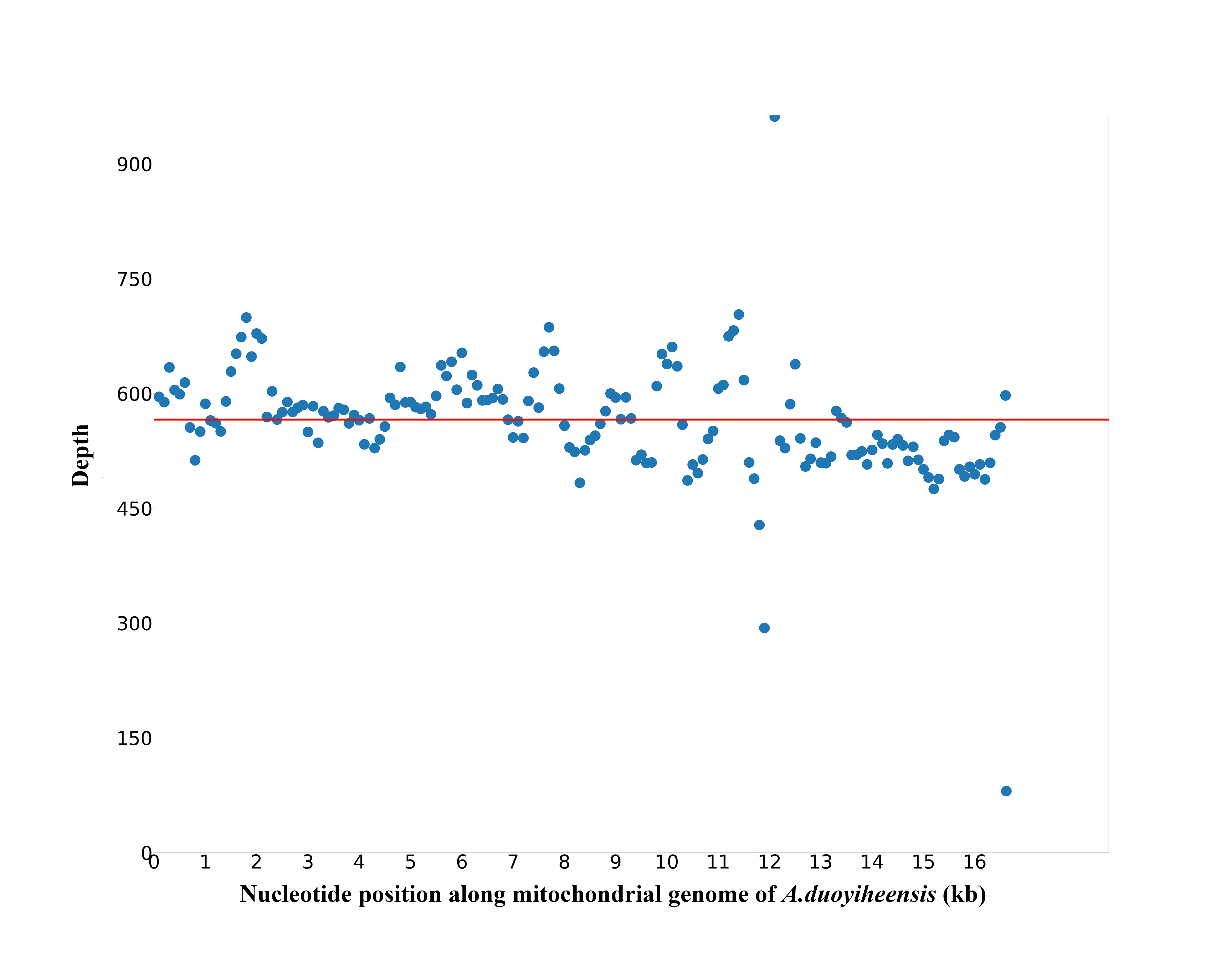

Supplement: Supplemental Material [file TMDN_A_2254459_SM5910.tif]
