# Supplementary material for: The complete mitochondrial genome and phylogenetic analysis of Anabarilius duoyiheensis Li, Mao & Lu, 2002 (Cypriniformes: Xenocyprididae)
Source: Mitochondrial DNA B Resour. 2023 Sep 20;8(9):989–92. doi: 10.1080/23802359.2023.2254459 (PMC10512799; doi:10.1080/23802359.2023.2254459)
Supplement: Supplemental Material [file TMDN_A_2254459_SM5809.docx]

Table S1. The assembly data statistics of ***A. duoyiheensis***

| **Item** | ***A. duoyiheensis*** |
| --- | --- |
| Total length (bp) | 16,614 |
| Total length without N (bp) | 16,614 |
| Total number | 1 |
| Gap number | 0 |
| GC content(%) | 46.23 |
| Type | Circle |
| Assemble data set (G) | 2.50 |
| Assemble reads pairs | 8,350,000 |
| Coverage | 253.61 |
| Average sequencing depth | 582.42 |
